# Regulation of cerebrovascular resistance below the lower limit of cerebral autoregulation during induced hypotension: an observational study

**DOI:** 10.1016/j.bja.2024.12.037

**Published:** 2025-02-28

**Authors:** Eline Kho, Rokus E.C. van den Dool, Sandjiv S. Mahes, Oskar T. Corsmit, Alexander P.J. Vlaar, Dave R. Koolbergen, Denise P. Veelo, Nicholaas H. Sperna Weiland, Rogier V. Immink

**Affiliations:** 1 Department of Anaesthesiology, Amsterdam UMC, University of Amsterdam, Amsterdam Cardiovascular Sciences, Amsterdam, the Netherlands; 2 Department of Intensive Care, Amsterdam UMC, University of Amsterdam, Amsterdam, the Netherlands; 3 Laboratory of Experimental Intensive Care and Anaesthesiology, Amsterdam UMC, University of Amsterdam, Amsterdam, the Netherlands; 4 Cardio-thoracic Surgery, Amsterdam UMC, University of Amsterdam, Amsterdam, the Netherlands

**Keywords:** arterial pressure, cerebral autoregulation, cerebrovascular resistance, induced hypotension, middle cerebral artery blood velocity, transcranial Doppler ultrasonography

## Abstract

**Background:**

To maintain adequate perfusion, cerebral blood flow (CBF) is preserved by changes in cerebrovascular resistance (CVR) inversely related to fluctuations in mean arterial blood pressure (MAP). It has been hypothesised that during progressive hypotension, a lower limit of cerebral autoregulation (LLCA) is reached beyond which cerebrovascular dilation becomes exhausted and CBF starts to decrease together with BP. We tested this hypothesis by assessing CVR above and below the LLCA.

**Methods:**

Radial arterial pressure, thermodilution cardiac output (CO), and mean middle cerebral artery blood velocity (MCAV_mean_) were recorded during sustained intraoperative hypotension clinically needed for off-pump aortic root aneurysm surgery. For each participant, the individual LLCA was determined. Systemic vascular resistance (SVR) and CVR were calculated, and changes below and above the LLCA were assessed with a generalised linear effect models.

**Results:**

For 50 participants undergoing aortic root surgery who met inclusion criteria, LLCA was located at 58 (12) mm Hg, with a corresponding MCAV_mean_ of 32 (8) cm s^−1^ and CO of 5.1 (1.2) L min^−1^. Above the LLCA, the decline in CVR and SVR were similar, both with 19% per 10 mm Hg decrease in MAP (*P*<0.001). Below the LLCA, CVR declined at a lower rate (7% per 10 mm Hg), whereas the decrease in SVR was 13% per 10 mm Hg decrease in MAP (both *P*<0.001).

**Conclusions:**

The continuing decline of CVR below the LLCA indicated that brain vasculature is still able to react on changing BP. This implies that LLCA should not be regarded as a fixed point but rather a transitional zone between exhausted and normally functioning autoregulation.

**Editor's key points**

• A lower limit of cerebral autoregulation (LLCA) has been hypothesised beyond which cerebrovascular dilation becomes exhausted and cerebral blood flow decreases in concert with mean arterial pressure.
• In a study of 50 patients undergoing aortic root surgery with induced hypotension, the authors tested this hypothesis by assessing cerebrovascular resistance above and below the LLCA.
• Below the LLCA, cerebral blood flow was reduced to a lesser extent than often assumed indicating that brain vasculature is still able to react to changing blood pressure.
• Thus the LLCA should not be regarded as a fixed point, but rather a transitional zone between exhausted and normally functioning cerebral autoregulation.

Cerebral autoregulation involves a set of fast and slow physiological regulatory mechanisms that maintain cerebral blood flow (CBF) relatively constant by changing cerebrovascular resistance (CVR) to fluctuations in mean arterial blood pressure (MAP). It has been hypothesised that during progressive hypotension, a lower limit of cerebral autoregulation (LLCA) is reached where cerebrovascular dilation becomes exhausted and CBF starts to decrease together with blood pressure.^1^ This suggests that CVR will remain constant below the LLCA. The location of the LLCA varies inter-individually between MAPs of ∼40 and 90 mm Hg.^2^, ^3^, ^4^, ^5^

In general, MAP below the LLCA is considered undesirable and is therefore actively avoided during anaesthesia by interventions that maintain cardiac output (CO) and systemic vascular resistance (SVR). However, a clear consensus regarding the relation between hypotension and organ damage, specifically brain tissue damage, has not been reached.^6^, ^7^, ^8^ The significance of cerebral perfusion pressure (CPP) has been debated,^9^ and a study of patients with induced sustained severe hypotension did not report any neurological injury.^10^ Furthermore, the incidence of intraoperative hypotension is as high as 60%,^11^ depending on definitions, whereas the incidence of intraoperative stroke is very low.^8^ This questions whether vasodilation at the LLCA is indeed exhausted; we hypothesised that residual cerebral vasodilation still takes place below the LLCA. This might result in greater flexibility for physicians to manage blood pressure near the lower limit. To test this hypothesis, we monitored radial artery blood pressure and thermodilution-based CO together with middle cerebral artery (MCA) blood velocity during sustained hypotension needed for off-pump aortic root aneurysm surgery. With these data, we located the LLCA in each patient individually. Based on this patient-specific LLCA, we assessed the SVR and CVR changes above and below the LLCA.

## Methods

The study protocol was approved by the Institutional Ethics Committee of the Amsterdam University Medical Centers (W20_244#20.270). All patients provided written informed consent for the study.

### Personalised external aortic root support surgery procedure

Personalised external aortic root support surgery (PEARS) is an innovative procedure designed to prevent further enlargement of the aortic root and ascending aorta in patients with connective tissue diseases, such as Marfan or Loeys–Dietz syndrome. This technique offers a less invasive alternative to standard aortic root replacement, with no need for cardiopulmonary bypass. Based on CT scan data, a polyester sleeve is customised to fit the patient's aorta. After sternotomy, the surgeon separates the enlarged aortic root and coronary origins from the adjacent heart structures. The fragile wall of the dilated aortic root necessitates surgical dissection with minimal wall tension. Therefore, the MAP is decreased to ∼ 35 mm Hg to facilitate surgery around the aortic root and minimise risk of wall rupture and bleeding.^5^^,^^12^

### Setting and instrumentation

Standard monitoring included blood oxygen saturation, radial artery cannulation, ECG, and transcranial Doppler ultrasonography (TCD), and was applied upon arrival in the operating theatre. After induction of general anaesthesia with propofol (1.0–2.5 mg kg^−1^), S-ketamine (0–0.25 mg kg^−1^), sufentanil (0.1–0.5 μg kg^−1^), remifentanil (5–25 μg kg^−1^·h^−1^), lidocaine (0.5–1 mg kg^−1^), and rocuronium (0.5–1.2 mg kg^−1^), the trachea was intubated. Anaesthesia was maintained with infusion of propofol (1–4 mg kg^−1^ h^−1^) supplemented with an infusion of S-ketamine (0.1–0.4 mg kg^−1^ h^−1^) and remifentanil (5–15 μg kg^−1^ h^−1^). If necessary, blood pressure was supported with a continuous norepinephrine infusion (0–0.08 μg kg^−1^ min^−1^). A lung-protective mechanical ventilation regimen was initiated, targeting arterial carbon dioxide partial pressure ($Pa_{{\text{CO}}_{2}}$) of 5.33 kPa, and used an oxygen fraction that resulted in an arterial oxygen partial pressure ($Pa_{O_{2}}$) of ∼16 kPa.

The pulmonary artery was cannulated utilising a Swan-Ganz catheter (Edwards Lifesciences, Irvine, CA, USA), inserted via the right internal jugular vein, and continuous CO was computed with either the Vigilance II monitor or the HemoSphere (Edwards Lifesciences), its successor entailing similar algorithms. Central venous pressure (CVP) was obtained using the proximal part of the Swan-Ganz catheter, situated in the superior vena cava. Core body temperature was maintained at 36°C and end-tidal partial pressure of CO_2_ (PetCO_2_) from exhaled air was obtained (Normocap 200; Datex-Ohmeda, Helsinki, Finland) with a side-stream sampling capnograph. With the TCD probe (Compumedics DWL Germany GmbH, Singen, Germany), the proximal segment of the MCA was insonated unilaterally at a depth of 45–65 mm and fixed with a headband (DWL Diamon Probe Fixation System, Compumedics DWL Germany GmbH).

### Induction of controlled hypotension

Hypotension was induced before aortic root manipulation by decreasing the norepinephrine infusion rate and increasing the continuous remifentanil infusion rate. If these adjustments were insufficient, a morphine or clonidine bolus was administered, or less frequently nitroglycerine or esmolol was added to the regimen.

### Data analysis

Radial blood pressure and middle cerebral artery mean blood flow velocity (MCAV_mean_), measured with the TCD, measurements were stored digitally with a sampling frequency of 200 Hz for offline analysis. Data were preprocessed to remove artifacts and poor-quality data. Beat-to-beat blood pressure values were obtained from the radial artery catheter. MAP was calculated as the mean of the integral over the pressure beat, similar to deriving MCAV_mean_ from the flow velocity signal. CO was either extracted offline from the electronic patient data record, or stored on the monitoring device every 20 s, depending on the device. The technique behind the continuous CO calculation did not change. CVP was retrieved from the electronic patient data record every minute. Critical closing pressure (CrCP), the pressure at which vessel walls start to collapse, was calculated from MAP and diastolic (DAP) values of blood pressure and MCAV.^13^^,^^14^

(1) $$CrCP=MAP-\frac{MCAVmean}{MCAVmean-MCAVdiastolic}*\left(MAP-DAP\right)$$

The driving pressure for brain perfusion is approximated by the CPP as the difference between MAP and CrCP. Stroke volume was calculated dividing CO over heart rate (HR). For comparison, all signals (MAP, MCAV_mean_, CO, CVP, HR, CPP, and PetCO_2_) were resampled to 60 Hz. CVR was calculated by dividing MAP by MCAV_mean_. For the calculation of SVR, MAP was divided by CO. Both CVR and SVR were normalised by dividing the CVR or SVR by the corresponding resistance found at the patient-specific LLCA (∗100–100) to compare relative changes in both parameters during hypotension and normotension, distinguished by the LLCA.

### Individual lower limit of cerebral autoregulation

The LLCA for each patient was computed offline based on MAP and MCAV_mean_ data, as described^15^ and applied in previous work.^5^ In short, a static cerebral autoregulation curve was simulated for each data point, comprising a regression line and a horizontal reference line. For every potential cerebral autoregulation curve, the distance of this curve to each data point was calculated and averaged as the mean squared error (MSE). This procedure was conducted for all conceivable cerebral autoregulation curves. The patient-specific static cerebral autoregulation curve, and consequently the patient-specific LLCA, was determined as the median of the five cerebral autoregulation curves with the lowest MSEs. Patients were excluded if the discrepancy between the LLCA values from the five lowest MSE cerebral autoregulation curves exceeded 5 mm Hg. With the individual LLCA, differences in MCAV_mean_, CO, and both resistances above and below the LLCA were assessed.

### Possible confounders

As blood pressure was very low in the study population, the effect of CVP on CPP might become relevant. Therefore, the absolute and relative differences between CVP and MAP were assessed. Other possible confounders such as the PetCO_2_ and HR were assessed as well. When confounders showed significant changes at different MAP values, this was corrected for.

### Statistical analysis

All descriptive data were presented as median [first–third quantile] or mean (standard deviation [sd]), if applicable. All data analyses and statistics were performed using MATLAB (Version 2019b, The MathWorks Inc., Natick, MA, USA).

Statistical differences in the signals (MCAV_mean_, CO, PetCO_2_, and CVP) or parameters (CVR and SVR) at different blood pressures were calculated for every 10 mm Hg increase, with generalised linear mixed model or the Friedman test in case of nonparametric data, and corrected *post hoc* by Bonferroni correction. The Bonferroni-corrected *P*-value was 0.05/7=0.007, as there were seven tests per parameter; every 10 mm Hg, a value was tested with a range from 35 to 105 mm Hg for MCAV_mean_ and CO. To test whether parameters changed below or above the LLCA, the same statistic test was applied based on four tests per parameter (below the LLCA at –35, –25, –15, and –5 mm Hg, and above the LLCA at 5, 15, 25, and 35 mm Hg, where LLCA is situated at 0 mm Hg).

A sample size was calculated, based on findings in a previous study,^5^ where normalised MCAV_mean_ above and below the LLCA was used (mean: 0.9 and 0.8, with sd: 0.1). Combined with a significance level of 0.05 and power of 80%, this resulted in a sample size of 34 patients.

## Results

A total of 62 patients were included between July 2019 and December 2023 (Table 1). Of these, one patient was excluded because of an inadequate TCD window, three patients were excluded because of measurement errors, and eight patients because of CO storage problems. Data from 50 patients, 13 females, were available for analysis with a mean age of 43 (range: 16–68) yr, mean (SD) height of 188 (9) cm, and mean weight of 86 (14) kg.

**Table 1:** Patient characteristics. Data are presented as mean (sd) or as number for the total population (*n*=50), and for the population where the lower limit of the cerebral autoregulation (LLCA) was adequately detected (*n*=34). FTAAD, familial thoracic aortic aneurysm and dissection.

	Total patients included (*n*=50)	Included in LLCA analyses (*n*=34)
Female/Male	13/37	9/25
Age (yr), mean (range)	43 (18–68)	43 (18–64)
Weight (kg), mean (SD)	86 (14)	86 (13)
Height (cm), mean (SD)	188 (9)	188 (8)
BMI (kg m^−2^), mean (SD)	24.3 (3.8)	24.3 (3.5)
**Cause of progressive dilation aorta root**		
Marfan syndrome	18	11
Loeys–Dietz syndrome	3	2
FTAAD	13	9
Other/unknown	16	12
**Hypotension induced with (a combination of)**		
Clonidine	24	15
Nitroglycerine	9	6
Esmolol	4	2
Morphine	46	32

**Fig 1:**
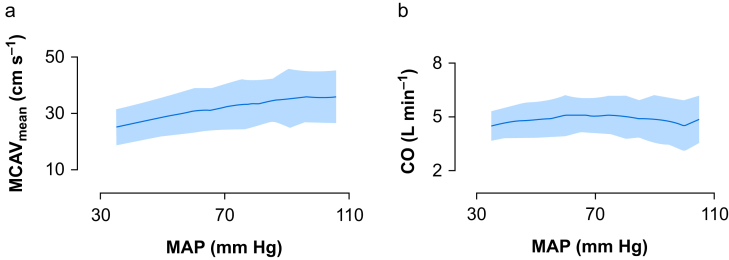
(a) Mean cerebral artery blood flow velocity (MCAV_mean_) and (b) cardiac output (CO) and plotted at different mean arterial blood pressures (MAPs), *n*=50. Statistical analyses were performed with a generalised linear mixed-effect model.

The trend of both CO and MCAV_mean_ showed an increase (with a generalised linear mixed model) for an increase in MAP; an increase of 10 mm Hg resulted in a CO increase of 0.03 L min^−1^ (*P*=0.021) and a MCAV_mean_ increase of 0.8 cm s^−1^ (*P*<0.001; Fig. 1). Applying the corrected Bonferroni *P*-value (*P*=0.007), CO was no longer significant.

### Lower limit of cerebral autoregulation

The individual LLCA findings are illustrated in Supplementary Figure S1. The LLCA could not be calculated for 16 patients (for seven patients, the average of the five lowest MSEs was >5 mm Hg, and for nine patients not enough data were acquired as recorded blood pressures were very low). With the remaining 34 patients, a mean (sd) LLCA of 58 (12) mm Hg was found, with a corresponding MCAV_mean_ of 32 (8) cm s^−1^ and CO of 5.1 (1.2) L min^−1^. The blood pressure was below the patient-specific LLCA for a mean (sd) duration of 93 (47) min during surgery.

### Applying the lower limit of cerebral autoregulation

**Fig 2:**
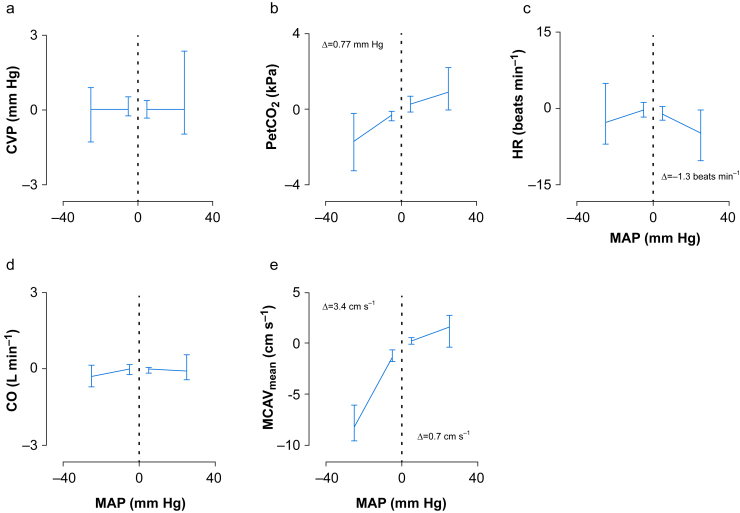
Parameters evaluated below and above the lower limit of cerebral autoregulation (LLCA). This limit is seen as the dotted line at 0 of the *x*-axis. The delta (Δ) increase or decrease of the parameters, calculated below and above the LLCA, are given in the figures, when significant (*P*<0.007, according to Bonferroni correction), calculated with generalised linear mixed-effect models (*n*=34). CO, cardiac output; CVP, central venous pressure; HR, heart rate; MAP, mean arterial blood pressure; MCAV_mean_, mean cerebral artery blood flow velocity; PetCO_2_, end-tidal CO_2_.

**Fig 3:**
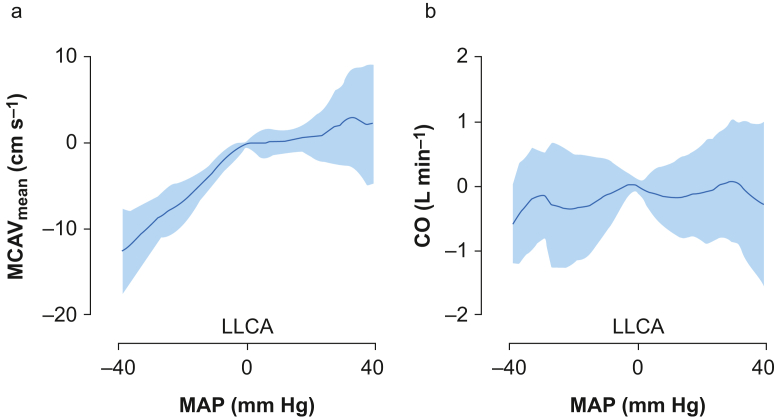
Trend in (a) mean cerebral artery blood flow velocity (MCAV_mean_) and (b) cardiac output (CO) at different mean arterial blood pressures (MAPs). MAP =0 mm Hg represents the individualised lower limit of the cerebral autoregulation (LLCA), with a mean (sd) of 58 (12) mm Hg based on 34 patients. Both MCAV_mean_ and CO are normalised by subtracting its value found at the LLCA.

With the individual LLCA of each patient, differences of parameters below and above the LLCA were assessed with the generalised linear mixed model. The overall mean (sd) estimated CPP was 28.7 (14.7) mm Hg, and significantly increased when blood pressure increased in both the hypotensive and normotensive blood pressure range (5.7 and 6.4 mm Hg per 10 mm Hg MAP increase, respectively, both *P*<0.001; Table 2). Stroke volume, with a mean (sd) of 74.0 (17.2) ml at the LLCA, did not show significant changes at different pressures. PetCO_2_ (mean [sd]: 5.07 [0.40] kPa) showed an overall significant increase of 0.07 kPa per 10 mm Hg MAP increase (*P*<0.001; Fig. 2 and Table 2). This was corrected for in subsequent MCAV_mean_ values by calculating the cerebrovascular CO_2_ reactivity for each subject, assuming a linear relationship in this range. Superimposing the data, using the individual LLCA of each patient, resulted in a characteristic Lassen curve (Fig. 3). Here, above the LLCA, MCAV_mean_ slightly increased (0.7 cm s^−1^) with increasing MAP with 10 mm Hg (*P*=0.006), but showed a greater decrease when MAP decreased further below the LLCA (with 3.4 cm s^−1^, *P*<0.001). Aside from the MCAV_mean_, the data were also superimposed for CO (Fig. 3), where CO increased by 0.09 L min^−1^ every 10 mm Hg above the LLCA (*P*=0.009, above the Bonferroni corrected *P*-value), but did not show a change below the LLCA.

**Table 2:** Haemodynamic changes below and above the lower limit of the cerebral autoregulation (LLCA). All statistical analyses were performed with a generalised linear mixed-effect model, based on 34 patients, except for the central venous pressure (CVP), which was calculated with Friedman test based on 30 patients. CO, cardiac output; CPP, cerebral perfusion pressure; CVR, cerebral vascular resistance; HR, heart rate; MCAV_mean_, mean cerebral artery blood flow velocity; PetCO_2_, end-tidal CO_2_; SV, stroke volume; SVR, systemic vascular resistance. ∗Corrected for PetCO_2_. ∗∗Bonferroni corrected *P*=0.007.

	Below LLCA	P-value∗∗	Above LLCA	P-value∗∗
CO (L min^−1^)	0.09	0.009	-0.009	0.840
MCAV_mean_ (cm s^−1^)	3.4∗	**<0.001**	0.7∗	**0.006**
PetCO_2_ (kPa)	0.10	**<0.001**	0.03	0.045
HR (beats min^−1^)	0.89	0.032	-1.3	**0.005**
CVP (mm Hg)	0.2	0.698	0.8	0.074
CPP (mm Hg)	5.7	**<0.001**	6.4	**<0.001**
SV (ml)	0.0	0.974	1.6	0.045
CVR (%)	7	**<0.001**	19	**<0.001**
SVR (%)	13	**<0.001**	19	**<0.001**

The overall median (first–third quantile) CVP was 3.6 (2.3–5.3) mm Hg based on 30 patients, and showed no change below or above the LLCA, calculated with the Friedman test (Fig. 2). Differences in PetCO_2_ and $Pa_{{\text{CO}}_{2}}$ over time showed no major discrepancies with a mean (sd) of –0.32 (1.13) kPa.

### Systemic and cerebrovascular resistance

**Fig 4:**
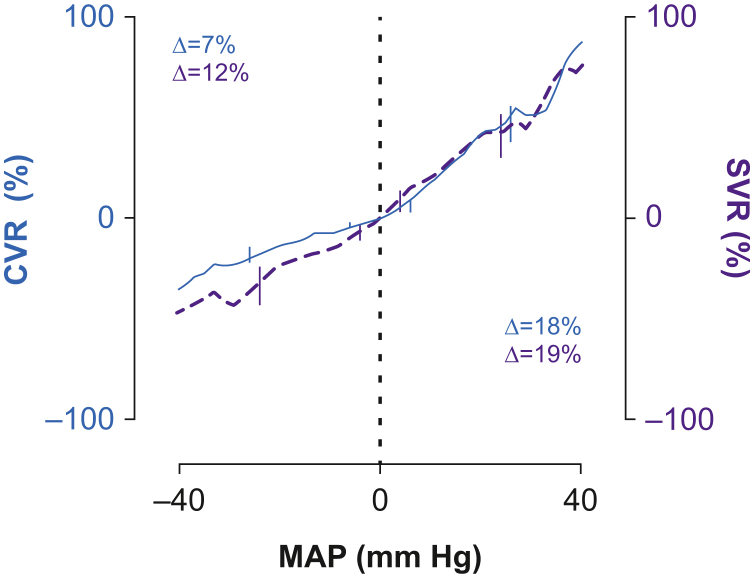
Systemic vascular resistance (SVR) and cerebral vascular resistance (CVR) shown in purple and blue, respectively. The (significant) increases in both parameters are tested with a generalised linear mixed-effect model below and above the lower limit of cerebral autoregulation. This limit is seen as the dotted black line at 0 on the *x*-axis. MAP, mean arterial blood pressure. (For interpretation of the references to color in this figure legend, the reader is referred to the Web version of this article).

Above the LLCA, an increase of 10 mm Hg resulted in an increase of 19% (95% confidence interval [CI]: 16–22%) in SVR, and 19% (95% CI: 15–23%) in CVR (both *P*<0.001; Fig. 4), calculated with the generalised linear mixed model. Below the LLCA, during hypotension, SVR decreased with 13% (95% CI: 12–14%) per 10 mm Hg decrease, whereas CVR showed a decrease of 7% (95% CI: 6–7%), both significant (*P*<0.001).

## Discussion

We showed that above the LLCA, where cerebral autoregulation is present, SVR and CVR paralleled each other during fluctuations in blood pressure. Below the patient-specific LLCA, there is still some residual cerebrovascular dilatory capacity. However, the parallel changes in SVR and CVR progressively diminish. This suggests that cerebral autoregulation should not be considered as a system with an on/off switch located at the lower limit, but as a system that gradually detoriates as blood pressure declines.

The classic cerebral autoregulation curve was proposed by Lassen in 1959,^1^ introducing an autoregulatory plateau, where CBF remains constant during blood pressure changes by cerebral vasoregulation. It was hypothesised that systemic vasoconstriction was followed by cerebral vasoconstriction and that systemic vasodilation resulted in cerebral vasodilation. Below the lower limit, cerebral autoregulation was no longer able to compensate, as CVR is exhausted and cerebral vessels are maximally dilated, and as a result CBF passively followed changes in blood pressure.^16^ Through the next decades, a few refinements to this model have been proposed.

Firstly, a perfectly flat plateau would require a near-infinite feedback gain not normally encountered in biological systems.^17^^,^^18^ Instead, it is likely that the plateau has a slope in the range of 0.5–4% in CBF per mm Hg.^17^ Secondly, fluctuation in the accuracy of cerebral autoregulation was observed in the plateau region.^5^^,^^19^^,^^20^ Thirdly, the position of the lower limit of the autoregulation shows inter-individual variation between MAPs of ∼40 and 90 mm Hg, comparable with other studies.^2^, ^3^, ^4^, ^5^ Additionally, our study challenges the original definition of the LLCA, as we could not confirm exhausted vasodilation below this point. Perhaps, the definition of the LLCA should not focus on the moment where vasodilatation is exhausted, but rather focus on the moment CVR no longer parallels SVR changes. From a clinical perspective, the outcome of our study suggests that reaching the LLCA is not a clear cutoff, so perfusion will not suddenly drop significantly below this point, providing a greater margin of safety.

The relation between neurological injury and blood pressures below the lower limit is not clear, as studies show different outcomes.^6^^,^^8^^,^^21^ For patients undergoing the PEARS procedure, and also in our study, no neurological injury was reported, although there are no reports of how this was determined.^10^^,^^22^^,^^23^ The absence of an association between hypotension and neurological injury might be explained by several factors. Firstly, the brain is able to adapt by increasing oxygen extraction from the blood.^24^^,^^25^ Secondly, when anesthetised, the CBF oxygen supply/demand ratio improves as a result of the reduced cerebral metabolic rate of oxygen, compensating for the effect of reduced CBF owing to propofol,^26^ resulting in a state of luxury perfusion.^27^ In our study, hypotension was desired and induced by vasodilation, and CO was maintained.

### Confounders

An important mechanism for CBF regulation is extracellular pH, largely determined by dissolved arterial CO_2_, which acts as a potent vasoactive signal in cerebral vessels. Local decreases in pH usually signify increased metabolism or local hypoxia, which is a signal for the brain to direct blood from less active tissues to brain areas were oxygen demand is high. Because acute changes in $Pa_{{\text{CO}}_{2}}$ lead to acute changes in pH, this mechanism has been referred to as cerebrovascular CO_2_ reactivity.^28^, ^29^, ^30^ In general, hypercapnia results in cerebral vasodilation and will increase CBF, whereas hypocapnia results in vasoconstriction and will decrease CBF. Between a $Pa_{{\text{CO}}_{2}}$ of 2.7–4.0 kPa per 0.5% kPa, CBF changes proportionally and more or less linearly by ∼ 0.5% per kPa.^31^ Beyond these boundaries, the relationship begins to resemble an exponential regression model or sigmoidal curve.^32^ Maximal cerebrovascular dilation probably occurs at $Pa_{{\text{CO}}_{2}}$ of ∼ 20.0 kPa (resulting in flow increases up to 240% of normal), and maximal vasoconstriction at a $Pa_{{\text{CO}}_{2}}$ of 0–1.3 kPa (resulting in flow reduction up to 60%).^33^

Our results suggest that variation in CBF is independent of the CO across a wide range of blood pressures. Variation of flow is explained by changes in blood pressure below the LLCA. Our results further show that, below the LLCA, changes in CBF are affected by SVR, as changes to MAP do not show a one-to-one relationship with the CBF. Instead, below the LLCA, when decreasing the pressure four times corresponds to a six times reduction of CBF. PetCO_2_ did not explain the CO–CBF nor the MAP–CBF relationship. One likely mediating mechanism is linearly increasing SVR with increasing pressure in the face of relatively constant CO. In addition, CVR seems to continue to decrease slightly below the LLCA. Both responses of SVR and CVR below the LLCA result in a disproportional preservation of CBF. In short, we found contradictory evidence for the classical, but increasingly questioned,^9^^,^^34^ lower limit of the Lassen curve, which dictates that below the LLCA there is a one-to-one change in the CPP–CBF relationship. This mechanism might be affected more by SVR and CVR than previously assumed.

### Strengths and limitations

The primary strength of this study is the availability of gold-standard CO measurements across a wide range of blood pressures in generally healthy individuals. We could therefore calculate CO and SVR, and study these interactions in more detail than previously possible in humans. Below the LLCA, CO exhibited an increase of 0.09 L min^−1^ per 10 mm Hg, which was not statistically significant. Additionally, an increase of 0.09 L min^−1^ per 10 mm Hg has negligible clinical implications. However, there are some limitations in this observational study. Firstly, our findings apply during general anaesthesia and not to awake humans. General anaesthesia induces a variety of haemodynamic changes (in part owing to sympathetic attenuation)^35^^,^^36^ and reduction of metabolism.^27^ All patients received a propofol-based anaesthesia regimen to enhance interpretation of intraoperative EEG. Propofol generally lowers CBF, but maintains cerebral autoregulation independently of dose.^20^^,^^37^ Similarly, to achieve deep sustained hypotension, reducing continuous administration of norepinephrine and increasing continuous remifentanil administration was not always sufficient to reach safe aortic transmural pressures for surgical manipulation; morphine, clonidine, and sometimes nitroglycerine or esmolol were administered to further reduce MAP. CBF is not affected by NTG^38^^,^^39^ or morphine,^40^ but for clonidine a relation was found with CBF, with a 23% decrease in flow after the administration of clonidine 5 μg kg^−1^.^41^ However, this was in awake patients, and in our study <1.6 μg kg^−1^ was administered in 15 patients, diminishing a possible clonidine effect on overall CBF calculations.

Secondly, intracranial pressure (ICP) was not measured. However, ICP is generally low in the healthy population in the supine position, and data are scarce. One study suggested that median ICP in this population is between –3 and 7 mm Hg, and should remain low if no positional changes are made to the head.^38^ Clinically, no signs of increased ICP were evident. Across all patients during the entire surgery, mean CVP did not change at different pressures, so CVP was omitted from CVR calculation because of its generally low value and variation, and the absence of invasive ICP. Furthermore, when calculating the CVR, it would be logical to use the CPP instead of a general MAP. However, as the CPP was not directly measured, the calculated CPP is a derivative, and therefore based on several assumptions. Implementing CPP instead of the MAP might therefore bring its own disadvantages. Additionally, as MAP is often used in other studies, for comparison reasons the MAP was used.

Conclusions regarding CBF using TCD-derived MCAV_mean_ should be considered in light of this method's limitations. Changes in MCAV_mean_ estimate changes in global CBF under the assumption that MCA diameter does not change and that flow in the centre of the vessel is laminar.^42^ An early validation study showed that MCA diameter barely changed despite fluctuations in blood pressure or CO₂ during direct observation in 12 patients undergoing craniotomy.^43^ However, because most of these patients underwent surgery for subarachnoid haemorrhage, normal intracranial vasomotor function cannot be assumed. Recent studies in healthy subjects using MRI to measure the diameter of the proximal MCA segments suggest that modest vessel diameter changes occur during hypocapnia, hypercapnia, and rhythmic hand grip exercises.^44^, ^45^, ^46^ Although these studies reject the notion that MCA diameter is fixed, the circumstances under which these changes were found do not apply to the current study. Specifically, this study maintained normocapnia throughout the interventions. Furthermore, the change in MCA diameter during hand grip exercises is assumed to be mediated by sympathetic activity, which is dramatically reduced or even absent during general anaesthesia.^47^, ^48^, ^49^ Other studies have shown that changes in MCAV_mean_ accurately reflect changes in CBF.^50^^,^^51^ In summary, we regard MCAV_mean_ as an acceptable proxy for estimating changes in CBF.

The LLCA was treated as a clear cutoff point as defined by Lassen. However, as suggested in this study, the position of the LLCA might not be fixed. Considering the LLCA more as a gradually decreasing point would affect the position of the LLCA. However, this would not have affected the trend of the CVR or SVR.

## Conclusions

We found that below the lower limit of cerebral autoregulation, cerebral blood flow is not entirely pressure-passive, and that the SVR is probably an important contributing mechanism to dampen reductions in cerebral blood flow. This implies that below the lower limit of cerebral autoregulation, cerebral blood flow is reduced to a lesser extent than often assumed.

## Authors' contributions

Study concept and design: EK, NSW, RI

Data acquisition: EK, RvdD

Data analysis and interpretation: DK, NSW, RI

Drafting of the manuscript: DK, NSW, RI

Critical revision of the manuscript: all authors

## Declaration of interest

APJV reports having received grants and consultancy fees from Edwards Lifesciences and Philips Medical BV. DPV reports having received grants and consultancy fees from Edwards Lifesciences and Philips Medical BV. RVI reports having received grants from Edwards Lifesciences.
