# Supplementary material for: Regulation of cerebrovascular resistance below the lower limit of cerebral autoregulation during induced hypotension: an observational study
Source: Br J Anaesth. 2025 Feb 28;134(4):1009–17. doi: 10.1016/j.bja.2024.12.037 (PMC11947574; doi:10.1016/j.bja.2024.12.037)
Supplement: Multimedia component 1 [file mmc1.docx]

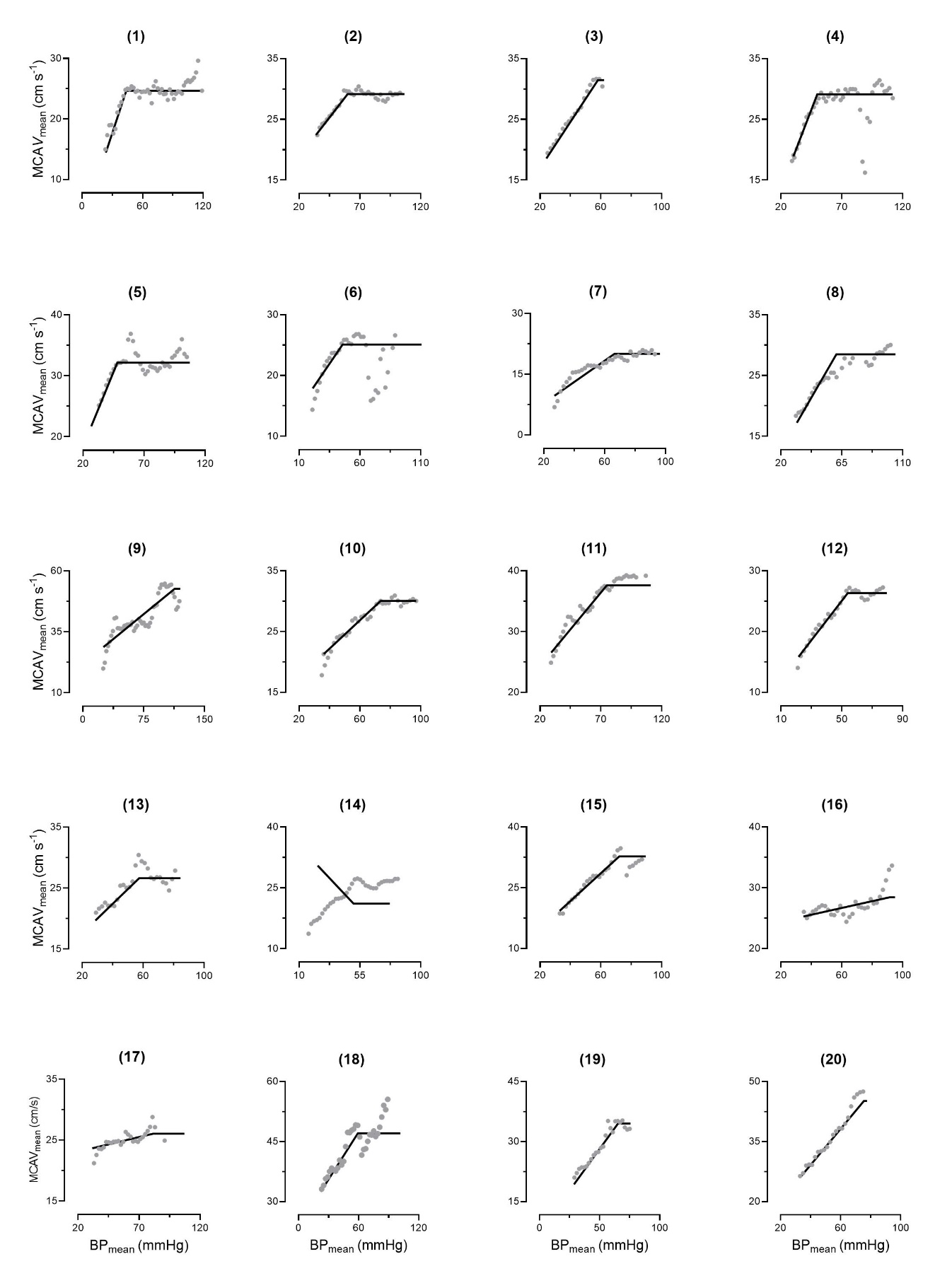


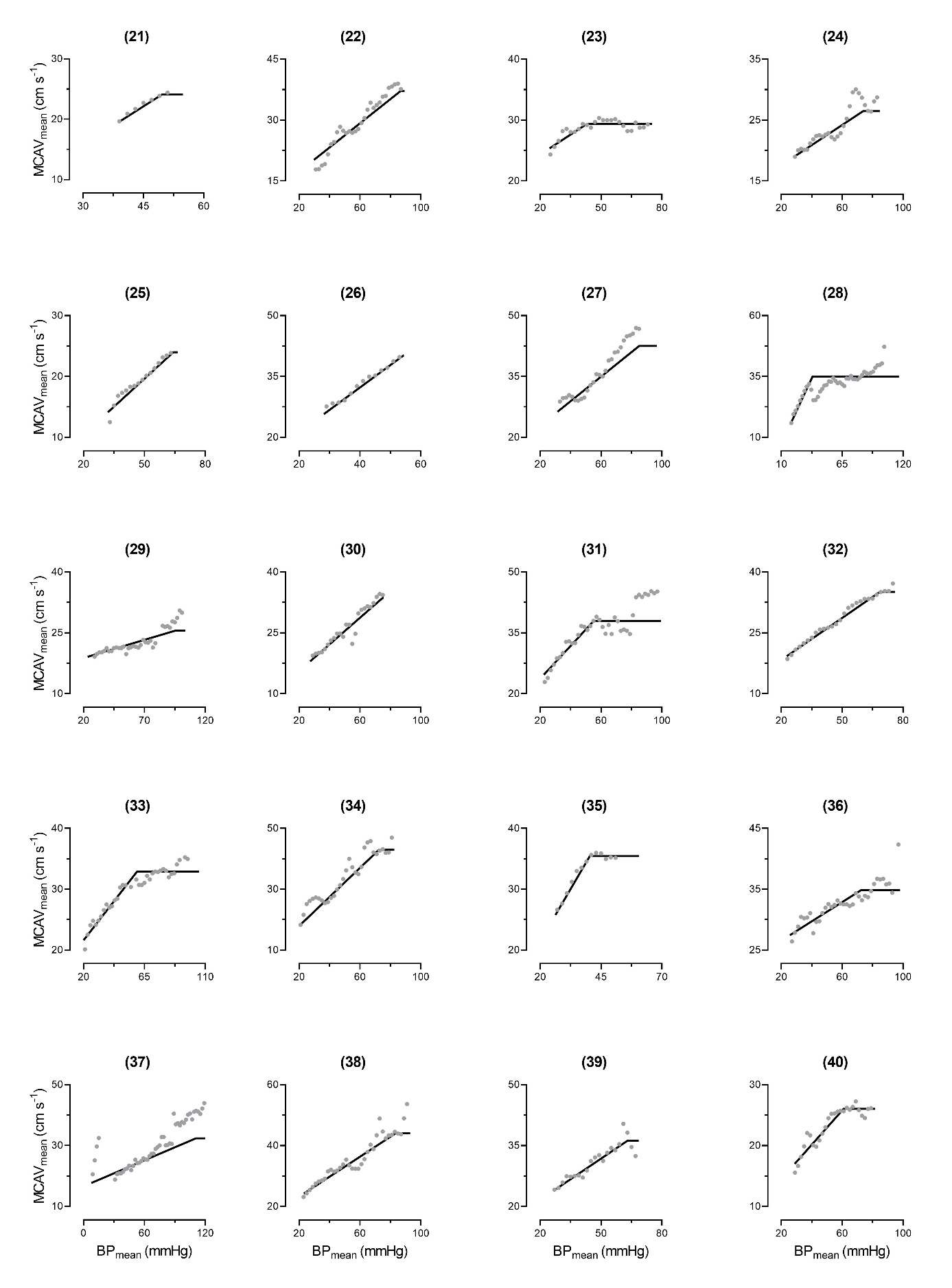


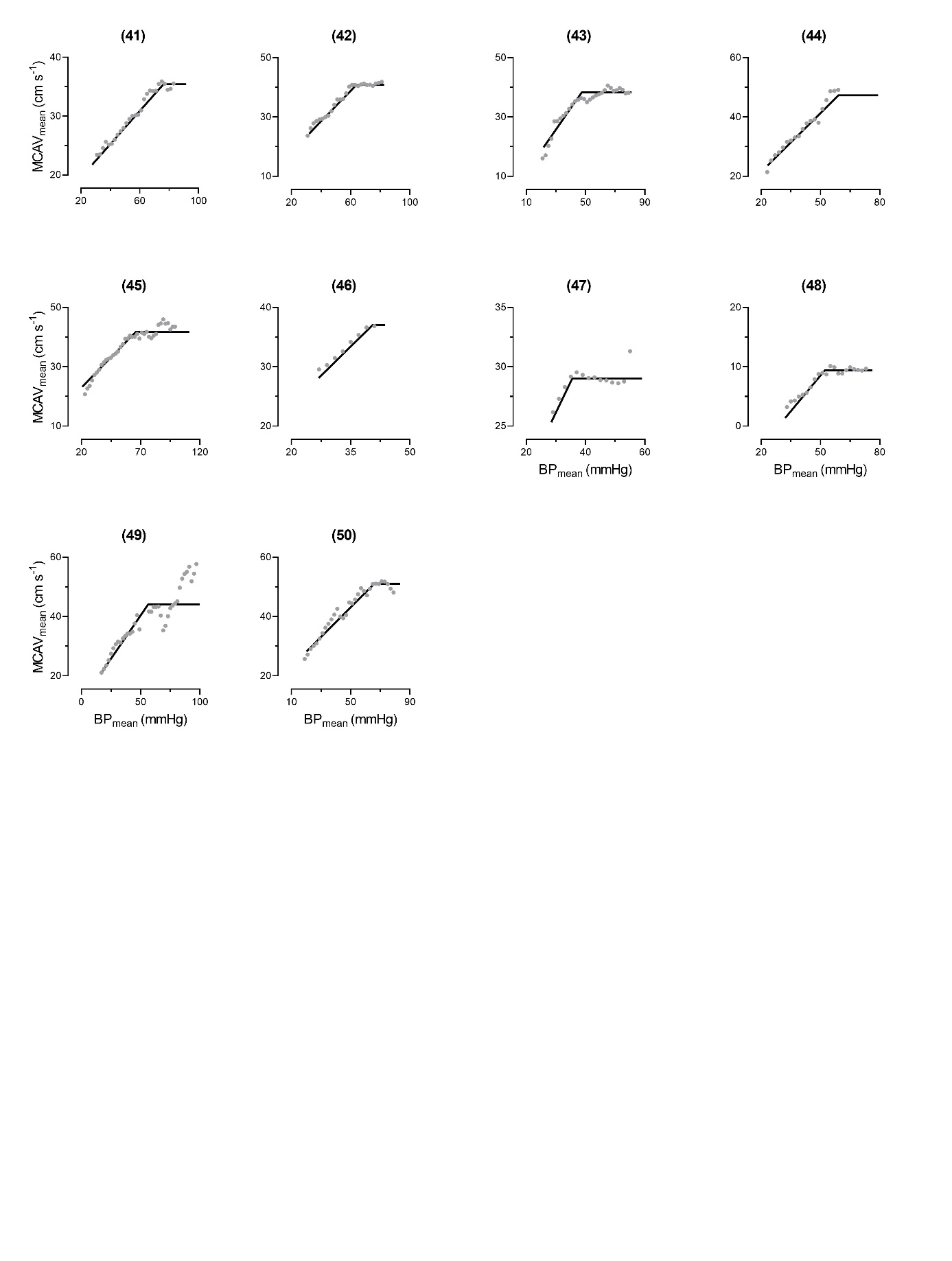


**Supplementary Fig S1** For 50 patients the static lower limit of the cerebral autoregulation (LLCA) was calculated according to the method of Schmidt and colleagues and represented as the black lines^15^. For visualization, mean arterial blood pressure data, MAP, and the corresponding mean cerebral arterial blood flow velocity (MCAV_mean_), was averaged per 2 mm Hg. Patients 9, 14, 16, 20, 22, 24-27, 29,30, 33, 36-38 and 56 were not implemented for further LLCA research. Here, patient 14 shows an expected averaged MCAV_mean­_ over MAP (grey dots), but the method of Schmidt was unable to correctly derive the corresponding curve with its LLCA, excluding this patient for further analyses.
